# Harm of circadian misalignment to the hearts of the adolescent wistar rats

**DOI:** 10.1186/s12967-022-03546-w

**Published:** 2022-08-06

**Authors:** YunLei Wang, YuanYuan Hou, ShaoFei Song, Yao Zuo, Yan Yu, YaFei Chi, Tong Zhang

**Affiliations:** 1 School of Rehabilitation Medicine, Capital Medical University, Beijing, 100068 China; 2 Beijing Bo’ai Hospital, China Rehabilitation Research Center, No.10 JiaoMen North Road, Fengtai District, Beijing, 100068 China; 3 China Rehabilitation Science Institute, Beijing, 100068 China; 4 Lab of Brain Injury Repair and Rehabilitation, China Rehabilitation Science Institute, Beijing, 100068 China; 5 Capital Medical University, Beijing, 100069 China; 6 Cheeloo College of Medicine, Shandong University, Jinan, 250012 Shandong China; 7 University of Health and Rehabilitation Sciences, Qingdao, 266071 Shandong China

**Keywords:** Circadian misalignment, Adolescent rats, Heart, Blood pressure, Clock gene

## Abstract

**Purpose:**

The purpose of this study was to observe the harm of circadian misalignment (CM), caused by an inverted photoperiod (IP), on the hearts of the adolescent Wistar rats, and to explore the mechanisms leading to harm.

**Methods:**

An IP was used to create a CM model. A total of 174 Wistar rats were randomly divided into circadian alignment (CA) and CM groups (87 rats per group). The different activity rhythms of the two groups of rats were adjusted through different light/dark cycles for 90 days. We recorded the rhythmic activity trajectory and sleep time of the rats. After 90 days of modeling, we performed various analyses (i.e., blood pressure, weight, cardiac ultrasound tests, serological tests, cardiac tissue immunofluorescence, immunohistochemistry, transmission electron microscopy on myocardial mitochondria, western blotting, and quantitative polymerase chain reactions).

**Results:**

(1) The IP protocol caused CM in rats. (2) CM rats showed significantly higher blood pressure during the day (resting phase). They also showed significantly higher serum levels of angiotensin II and epinephrine during the day compared to the CA rats. (3) CM caused up-regulation of gene expression of adrenergic receptors α1 (*α1-AR*) and β1 (*β1-AR*) and down-regulation of the glucocorticoid receptor (*Gr)* gene expression in rat hearts. It also caused downregulation of *Bmal1* expression. In addition, the changes in *Bmal1* and *Per2* correlated with the changes in *β1-AR* and *α1-AR*. (4) CM had adverse effects on multiple molecular proteins of the heart. (5) CM increased the collagen fibers in the rat heart and increased the destruction of mitochondria. (6) Eventually, CM caused a decrease in the pumping function of the heart and decreased the coronary blood flow rate.

**Conclusions:**

(1) CM significantly affected the cardiac structure and function in the adolescent rats through a variety of mechanisms. (2) CM can regulate the expression of myocardial clock genes, and it is likely to have an impact on the heart through this pathway.

**Supplementary Information:**

The online version contains supplementary material available at 10.1186/s12967-022-03546-w.

## Introduction

Rapid economic expansion and development have made the phenomenon of circadian misalignment (CM) more common. Many people feel palpitations or chest tightness after shifting work schedules or staying up late [1, 2]. In addition, published clinical studies in humans show that CM can induce cardiovascular disease by increasing blood pressure, increasing serum inflammatory factors, and interfering with the normal heart rhythm [3]. Unfortunately, due to the lack of research on related mechanisms and specific effects of CM, many still have insufficient awareness of the great harm it can cause. With CM caused by shift work and jet lag showing an increasingly common trend [4], most people still ignore its potential for harm. The danger caused by CM to the heart is still regarded to be unlikely.

CM increases the incidence of myocardial infarction in the shift workers by promoting myocardial ischemia [5–7]. Furthermore, under similar conditions of CM, the probability of cardiovascular disease is lower in young individuals including adolescents compared to the middle-aged adults and the elderly [5, 8]. Currently, the adverse effects of CM on the hearts of young individuals is not clear. Several clinical studies have demonstrated that CM significantly increases the risk of cardiovascular disease in the young individuals [9]. However, heart disease is not reported in majority of the young and middle-aged working individuals who are diagnosed with CM [10]. Therefore, the underlying risk of cardiac dysfunction is not known for a large proportion of young people diagnosed with CM. In particular, the effects of CM on adolescents have not been reported. Furthermore, preventive therapy for CM is not well developed because the underlying mechanisms have not been well established. Therefore, in this study, we generated the Inverted photoperiod (IP)-induced CM model in the adolescent Wistar rats and investigated the underlying mechanisms that are responsible for the adverse changes in the heart structure and functions.

IP activity is the most common type of CM and is mainly dependent on exogenous entrainment [11]. Illumination triggers the activation of visual conduction pathways, which transmit signals to the suprachiasmatic nucleus (SCN) of the hypothalamus. SCN synchronizes the physiological functions of all the tissues and organs in response to the external environment by modulating the neuronal and humoral conduction pathways [12, 13]. The IP protocol described previously was used to induce CM in the normal 5-week-old Wistar rats for 90 days [14]. Then, the effects of CM on the cardiac structure and function of the adolescent Wistar rats and the underlying mechanisms were investigated.

## Methods

### Experimental animals

According to results from preliminary experiments, 174 male, 5-week-old, Wistar rats were selected and randomly divided into two groups, namely the circadian alignment (CA) and CM groups (87 rats per group). All rats were purchased from Weitong Lihua Co., Ltd. (Weitong Lihua Co., Ltd., Beijing, China) and raised in a specific, pathogen-free laboratory without specific bacterial barriers at Capital Medical University (Beijing, China). The breeding room was illuminated by 100 W fluorescent lamps, and the light intensity was 150–200 Lu. The indoor temperature was constant at 20–24 ℃, and the air humidity was 40–70%. The rats were fed food and water ad libitum. The food for all rats was conventional “rat maintenance feed” (Beijing Keao Xieli Feed Co., Ltd., Beijing, China). The raw material composition of the food mainly included corn, beans, and flour; the fat content was < 40 g/kg, and the caloric value was 3.4 kcal/g.

All animal protocols used in this experiment were reviewed and approved by the Ethics Committee of Capital Medical University (AEEI-2019–185) and were in accordance with the International Guiding Principles for Biomedical Research Involving Animals established by the World Health Organization (WHO).

### Light/dark modeling methods

All rats were reared under normal light/dark (LD) conditions for 1 week to adapt them to the experimental environment. Subsequently, the activity rhythm of the rats was adjusted through altering photoperiods. The LD cycle of the CA group (Additional file 1: Fig. S1B) was as follows: light time 08:00–20:00 and dark time 20:00–08:00 the next day. The LD cycle of the CM group (Additional file 1: Fig. S1B) was as follows: light time 08:00–20:00 and dark time 20:00–08:00 the next day, for a total of 3 days. Subsequently, this pattern was reversed to dark time 08:00–20:00 and light time 20:00–08:00 the next day for a total of 3 days. The two groups of rats were modeled in this manner for a total of 90 days.

After 90 days of modeling, the 174 rats were divided into three parts, namely part 1, part 2 and part 3(Additional file 1: Fig. S1C). In the part 1 group, there were a total of 90 rats (CA group: 45 rats; CM group: 45 rats). In this part, blood pressure was measured at two time points Zeitgeber (ZT)0 (08:00) and ZT12 (20:00) on day 91. Ultrasound measurements was measured at ZT0 (08:00) on day 92. Because the cardiovascular diseases often occur in the early morning (light/dark alternating time), and the blood pressure changes greatly at the light/dark alternating time [15, 16] so this experiment selected two time points (08:00 and 20:00) as a special collection point. The rats were sacrificed at either 08:00 or 20:00 on the 93rd day. Serum was extracted for Enzyme-linked immunosorbent assay (Elisa) tests and heart tissue was extracted for morphological examination and western blotting (WB) analysis.

In the part 2 group, there were a total of 60 rats (CA group: 30 rats; CM group: 30 rats). A random allocation of 5 CA rats and 5 CM rats from part 2 were sacrificed at each of six different time points on days 91–92. Then, serum was immediately extracted for ELISA tests and myocardial tissue was extracted for and quantitative polymerase chain reaction (qPCR) tests.

In the part 3 group, there were a total of 24 rats (CA group: 12 rats; CM group: 12 rats). Blood pressure was measured for all the part 3 rats at four time points, namely, ZT4 (12:00), ZT8 (16:00), ZT16 (24:00), and ZT20 (04:00) on day 91. The rats in this group were designated as the supplementary experimental group and only blood pressure was measured for these rats (see Additional file 1C).

### Clock lab behavioral analysis

The test equipment in this experiment consisted of a rat cage (45 cm × 25 cm × 20 cm) with a wheel as the basic unit and the American Clock Lab (ACT-500, USA) signal transduction instrument transmitting the data signal to the computer. On day 60 of this experiment, we randomly selected 10 rats from each of the CA and CM groups (20 rats in total). Each rat was subjected to behavioral analysis for 12 consecutive days. At the end of the experiment, a 24-h activity map of rats was formed using the Clock Lab (2.7.3) data package based on MATLAB (2014). The “Chi-squared Periodogram” data package was used to analyze the rhythm amplitude and rhythm period of each rat during these 12 days.

### HomeCage fine-behavior analysis

After 75 days of modeling, eight rats in each group were randomly selected (16 rats in total) and subjected to behavioral analysis for 6 days consecutively. The HomeCageScan system (CleverSys Inc, USA) was used to record the behavior of rats through a camera, and each rat was placed in a squirrel cage equipped with a camera. For video analysis, the supporting HomeCageScan™ 3.0. software (CleverSys Inc, Reston, USA) was used to obtain information regarding behavioral sleeping, eating, and other rest/activity time charts corresponding to the 24-h cycle of rats. The sleep hour and duration of the two groups of rats were determined. The average sleep time of each rat at 16:00 (i.e., length of sleep at 16:00) on the fifth day of the HomeCage test period (CM rats were under the first LD light on that day) was statistically analyzed, and the average sleep time at 16:00 = 15:00–17:00 total sleep time/3.

### Blood pressure testing

The LD cycle required 6 days in our experiments (Additional file 1: Fig. S1). The rats completed 15 LD cycles during the 90-day modeling period. Therefore, the phase of the CM and the CA rats was similar on days 91–93. Blood pressure was measured on day 91. Since blood pressure varies between day and night, it was measured for the 48 randomly selected part 1 rats at 08:00 (ZT0; CA:12 rats, CM:12 rats) and 20:00 (ZT12; CA:12 rats, CM:12 rats). Furthermore, blood pressure of the 24 rats in part 3 was measured at four time points—12:00 (ZT4), 16:00 (ZT8), 24:00 (ZT16), and 04:00 (ZT20).

To measure blood pressures in the rats, a six-channel rat non-invasive CODA sphygmomanometer (KENT, USA), was used. The systolic blood pressure, diastolic blood pressure, and mean arterial pressure were recorded using the rat-tail artery measurement method.

### Weight

This analysis was carried out prior to starting the experiments and again on day 91 of the experiment at ZT1 (09:00). Ten rats from each group (20 rats in total) were randomly selected for the weight test.

### Rat heart ultrasound

On day 92, ultrasonography of the rat hearts was performed. A total of 45 rats (CA group, 21 rats; CM group, 24 rats) were subjected to cardiac ultrasound examination (ZT0–2 08:00–10:00). A high-resolution, small-animal ultrasound imaging system (Vevo 2100; FUJIFILM VisualSonics, Inc., Bothell, Washington, USA) was used to perform rat heart measurements. Following anesthesia with isoflurane, the chest hair of rats was shaved on the test bench, and the M-mode was used to obtain the M-shaped motion curve of the left ventricle near the sternal bone of the rat heart. In addition, the structure and function of the left ventricle of the rats were scanned in multiple directions. Hemodynamic measurements were performed using color Doppler ultrasound to scan the coronary blood flow of the hearts and record blood flow velocity and coronary transvalvular pressure.

### Serum test and heart tissue test

On the 93rd day of this experiment, 60 rats (CA group:30 rats; CM group:30 rats) were randomly selected from the 90 rats in part 1 and sacrificed at ZT0 (08:00). The remaining 30 rats (CA:15 rats; CM: 15 rats) were sacrificed at ZT12 (20:00). Rats from part 2 (60 rats total) were sacrificed at one of six time points on days 91–92, after which blood was collected, and their hearts were taken. Ten rats were randomly sacrificed at each time point (CA group:5 rats; CM group: 5 rats). All blood was collected from the apex of the heart. After blood collection, the rats were sacrificed and myocardial tissue was taken. The allocated number of rats from which serum and heart tissue were taken for the different experiments is shown in Additional file 1: Fig. S1.

The experimental method was as follows: rats were deeply anesthetized using an intraperitoneal injection of 50 mg/kg of sodium pentobarbital. Surgical scissors were used to remove the ribs and expose the thoracic cavity. Blood was collected from the apex of the heart. Blood (2–3 ml) drawn from each rat was centrifuged after standing for 1–2 h. The upper layer of the serum was obtained and stored at − 80 °C for subsequent testing. Immediately after blood collection at the apex, the rat heart was separated and washed in normal saline at 4 °C. After flushing out the blood from the heart, the following experiments were performed:

1. Of the 30 rats from part 1 of the experiment, the hearts of 24 rats were randomly selected from each group (CM and CA) at ZT0 (08:00). The hearts were placed in a paraformaldehyde solution for fixation, and mounted in wax blocks for subsequent staining (i.e., Masson’s trichrome, Oil Red O, immunohistochemistry, and immunofluorescence).
2. The apical tissues (1 mm^3^) of the remaining 6 (out of 30) hearts of the rats from part 1 of the experiment were taken at ZT0 (08:00) and immediately placed in electron microscopy fixation solution for transmission electron microscopy (TEM) staining. The remaining apical tissues of hearts from six rats were placed in an Eppendorf tube, rapidly frozen in liquid nitrogen, and placed in a refrigerator at − 80 °C. The apical tissues of 5 rats were randomly selected for subsequent WB analysis.
3. Of the 60 rats from part 2 of the experiment, which were randomly sacrificed at one of six time points, the hearts were extracted and their apical tissues were placed in Eppendorf tubes, rapidly frozen in liquid nitrogen, and stored at − 80 °C for subsequent quantitative polymerase chain reactions.

### Enzyme-linked immunosorbent assay testing

This experiment was carried out as follows:

1. From the rats from part 1 of the experiment, the sera from rats sacrificed only at the single time point (08:00) were used to measure the concentrations of creatine kinase-MB (CK-MB; H197; Nanjing JianCheng, Nanjing, China; 24 rats from each of the CA and CM groups); lactate dehydrogenase (LDH; A020-2–2; Nanjing JianCheng; 30 rats from each of the CA and CM groups); and brain natriuretic peptide (BNP; EIAR-BNP-1; RayBiotech, Norcross, GA, USA; 30 rats from each of the CA and CM groups).
2. From the rats from part 1 of the experiment, sacrificed at one of two time points (08:00 or 20:00), the sera were measured for concentrations of angiotensin II (ANGII; EIAR-ANGII-1; RayBiotech, Norcross, GA, USA; 15 rats from each of the CA and CM groups at each of the 08:00 and 20:00 time points).
3. Rats from part 2 of the experiment, sacrificed at one of six time points groups (5 rats from each of the CM and CA groups at each time point), the sera were measured for concentrations of epinephrine (EPI, CEA858Ge), norepinephrine (NE, CEA907Ge), and glucocorticoid (COR, CEA462Ge). These three ELISA kits were all obtained from Cloud-Clone Corp. (Wuhan, China).

The specific number of rats in the two parts of the experiment are explained in Additional file 1: Fig. S1. The experimental method of Elisa test is in the Additional file 3: Experimental methods.

### Oil Red O staining of the myocardium (CA group: 20 rats, CM group: 22 rats)

Fresh frozen sections were placed into fixative and soaked in Oil Red O staining solution. After background differentiation, hematoxylin staining was performed, and the slides were mounted. The percentage of the cross-sectional area of the myocardium occupied by the Oil Red O color area was calculated. The lipid droplets were orange-red to bright red, and the nucleus of each cell was blue.

### Masson’s trichrome staining of the myocardium (CA group: 22 rats, CM group: 19 rats)

The paraffin sections were deparaffinized with water and placed in Masson A solution—a mixture of equal proportions of Masson B and Masson C solutions, Masson D liquid, Masson E liquid, and Masson F liquid. Next, glacial acetic acid was used to differentiate the sections. Finally, the sections were blocked. The blue color in tissues indicated the presence of collagen fibers. After the completion of staining, ImageJ software (National Institutes of Health, Bethesda, MD, USA) was used to calculate the proportion of collagen fibers: collagen fiber ratio = collagen fiber area/myocardial cross-sectional area.

### Immunohistochemistry

The immunohistochemistry in this experiment included the following antibodies: caspase-3 (CASP3; GB11009-1; Servicebio, Wuhan, China; 9 rats from each of the CA and CM groups); interleukin-6 (IL-6; GB11117; Servicebio; 13 and 14 rats from the CA group and CM groups, respectively); mammalian target of rapamycin (mTOR; GB11117; Servicebio; 13 rats from each of the CA and CM groups); nuclear factor-kappa B (NF-κB; 13,533–1- AP; Proteintech, Wuhan, China; 9 rats from each of the CA and CM groups); and tumor necrosis factor-alpha (TNF-α; bsm-33207 m; Bioss, Beijing, China; 13 and 14 rats from the CA group and CM groups, respectively). The experimental method of this part is in the Additional file 3: Experimental methods.

### Immunofluorescence

The myocardial tissue sections of the study rats were analyzed by immunofluorescence (IF) for the expression of CD34 (GB13013; Servicebio; 10 CA rats and 11 CM rats), heat shock protein 60 (HSP60; GB11243; Servicebio; 9 rats each from the CA and CM groups), Calcium Voltage-Gated Channel Subunit Alpha1 C (CACNA1C; 21,774–1-ap, Proteintech, Wuhan, China; 8 CA rats and 9 CM rats), and Coiled-Coil, Moesin-Like BCL2-Interacting Protein (BECLIN-1; GB112053, Servicebio; 8 CA rats and 9 CM rats). The IF protocol is described in the Additional file 3: Methods section.

### TEM of myocardial tissue

Six rats were selected from each group (12 rats in total), and the myocardium at the apex of the heart was obtained for TEM detection. The experimental method of this part is in the Additional file 3: Experimental methods.

The Flameng scoring method was used to evaluate the damage to the myocardial mitochondria of each rat (higher scores indicated more severe mitochondrial damage) [17].

### Western blotting

Five rats were randomly selected from each group (10 rats in total), and the myocardium at the apex of the heart was obtained for western blot detection. This experiment was performed to observe the relative expression of the following proteins in myocardial tissue: TNF-α (bsm-33207 m; Bioss), sprouty RTK signaling antagonist 2 (SPRY2; GB11860; Servicebio), receptor for advanced glycosylation end-product specific (RAGE; 16,346–1-AP; Proteintech), lamin B1 (GB1111802; Servicebio), lamin A/C (GB11407, Servicebio), IL-6 (GB11117, Servicebio) and CACNA1C (21774-1-ap; proteintech). The experimental method of this part is in the Additional file 3: Experimental methods.

### Quantitative polymerase chain reaction

The hearts of 60 rats were taken at 6 time points in part 2 (5 rats in each group at each time point), and the apical tissue was taken for qPCR. Each sample was measured in three repetitions, and the average CT value of the three repeated measurements was used for statistical analysis. Primers used were listed in Table 1. The experimental method of this part is in the Additional file 3: Experimental methods.

**Table 1:** Primer sequences

Gene	Search number	Forward	Reverse	Fragment length (bp)
GAPDH	NM_017008.4	CTGGAGAAACCTGCCAAGTATG	GGTGGAAGAATGGGAGTTGCT	138
Gr	XM_017600839.2	AGGTCTGAAGAGCCAAGAGTTA	TGGAAGCAGTAGGTAAGGAGAT	180
β1-AR	NM_012701.1	CTGGACTTCGGTAGACGTGC	CAGCACTTGGGGTCGTTGTA	249
α1-AR	NM_017191.2	ATTGGGTCTTTCTTCCCGGAT	AGGCTTTCTTGAACTCCTGGCT	130
Bmal1	NM_024362.2	GAGGCGTCGGGACAAAATGA	GCTTCTGTGTATGGGTTGGTGG	159
Clock	NM_021856.2	CTTACAGACATCTCGGTTGCTCC	TGGTGCGAAGGAGGGAAAGT	99
Per2	NM_031678.1	GCAGGCAGCAGTGATACAAGTC	CCTGCAAGACGTACTTAATGAACTG	130
Creb1	NM_031017.2	CATTGCCCCTGGAGTTGTTAT	CTCTTGCTGCTTCCCTGTTCTT	113

### Statistical analysis

Statistical analysis was performed using SPSS version 24.0 (IBM Corporation, Armonk, NY, USA) and R v.3.5.0 software. The statistical charts and data were visualized using Graphpad7.0 (GraphPad, La Jolla, CA, USA) and R v.3.5.0 software. Prior to statistical analysis, the data were tested for normality, and the two-sample *t*-test was used for normally-distributed data. Values are presented as the mean ± standard deviation. Non-compliance with the normal distribution was detected using the Wilcoxon rank-sum test and the interquartile range method, including the median.

The two groups of different time points were compared by an independent *t* test at the same time point. Correlation analysis was used to explore the correlation between factors. The "systematic clustering method" was used to draw a two-way heat map of myocardial genomics to explore the grouping of genes. The "Bayesian network diagram" was used to analyze the causal relationship of CM to gene expression (Parents-Descendants), and the heuristic mode was used to explore the causal relationship between genes.

The “MetaCycle package” [18] based on the R language was used to analyze the 24-h blood pressure biorhythm. The physiological effects of blood pressure were analyzed using the "Meta2d_pvalue". *p* < 0.05 indicated circadian rhythm characteristics for the blood pressure and *p* ≥ 0.05 indicated absence of circadian rhythm characteristics for the blood pressure. “Meta2d_AMP” was used to analyze the rhythmic amplitude of blood pressure, which indicated the strength of the circadian rhythm changes based on the changes of blood pressure. Meta2d_phase was used to analyze the rhythmic phase of blood pressure (the time point corresponding to the highest value in the circadian rhythm of the blood pressure).

## Results

### ClockLab and HomeCage behavior analysis

**Fig. 1:**
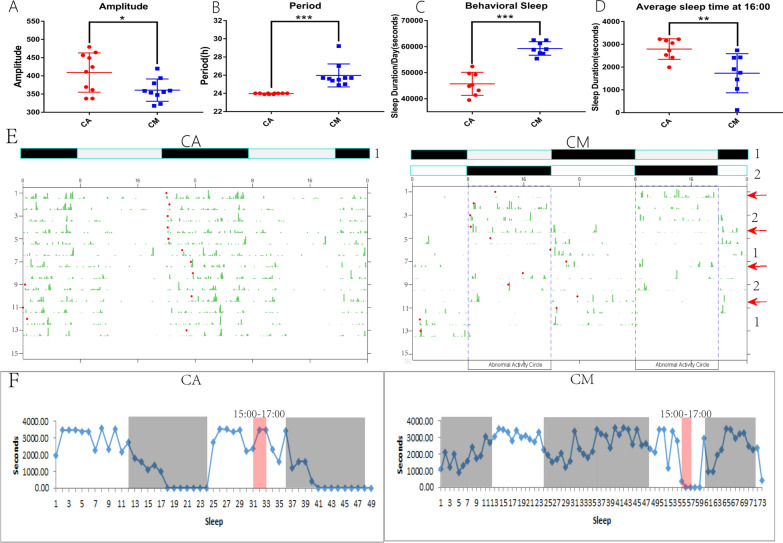
Rhythm amplitude (**A**) and rhythm period (**B**) of two different LD-cycle rats measured by Clock Lab. Twenty-four-hour sleep duration (**C**) and the average sleep duration at 16:00 (**D**) of rats with two rhythms measured by HomeCage. *p < 0.05, **p < 0.01, ***p < 0.001 (two-sample *t*-test). **E** The 24-h activity track of the two groups of rats over 12 consecutive days. The green mark represents the number of times the rat pushed the wheel at this time point, and the red dot represents the onset of the rat’s activity. IP prolonged the rhythm period of rats and gradually delayed the onset of activity. **F** The 24-h sleep duration trajectory of the two groups of rats. The CA group was measured for 2 days (48 h), and the photoperiod was LD. The CM group was continuously measured for 3 days (72 h), and the photoperiods were DL, DD, and LD. The black shading denotes that the rat was in the dark phase. The white area indicates that the rat was in the light phase. The area shaded in pink represents the sleep time of rats from 15:00 to 17:00, and the average sleep time of rat at 16:00. Abbreviations: CA, circadian alignment; CM, circadian misalignment; DD, dark/dark; DL, dark/light; IP, inverted photoperiod; LD, light/dark

Figures 1A and B present the results for the rhythm amplitude and period, respectively. The data showed that, compared with LD12:12, the ILD12:12 rhythm amplitude and period were significantly reduced (*p* < 0.05) and significantly longer (*p* < 0.001), respectively.

Figure 1C and D, respectively, represent the measured 24-h behavioral sleep time and the 16:00 average sleep time (15:00–17:00 average sleep time) statistical results. The results showed that, compared with CA group rats, CM rats had significantly longer periods of behavioral sleep in 24 h (*p* < 0.001). However, the average sleep time of CM rats at 16:00 (15:00–17:00 average sleep time) was significantly reduced (*p* < 0.01).

### Blood pressure analysis

**Fig. 2:**
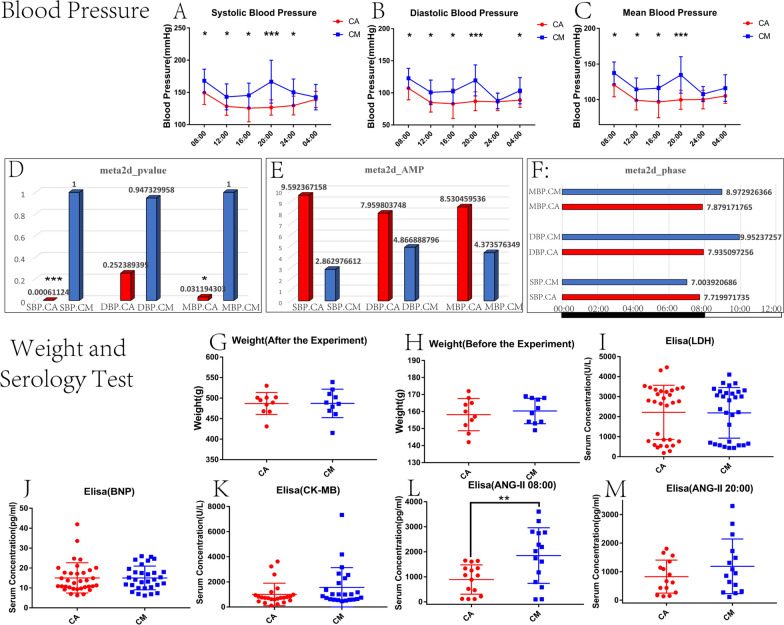
**A–C** The systolic blood pressure (**A**), diastolic blood pressure (**B**), and mean arterial pressure (**C**) of CM and CA rats at different time points. **D**–**F** The meta2d_pvalue (**D**), mata2d_AMP (**E**), and meta2d_phase (**F**) of the blood pressure in the CM and CA rats at six time points during the 24-h cycle, respectively. *p < 0.05, **p < 0.01, ***p < 0.001. Weights of rats in the two groups after (**G**) and before (**H**) the modeling. Serum LDH (**I**), BNP (**J**), CK-MB (**K**), ANGII (08:00) (**L**), and ANGII (20:00) (**M**) of the two groups of rats. *p < 0.05, **p < 0.01, ***p < 0.001 (two-sample *t*-test). Abbreviations: ANGII, angiotensin II; BNP, brain natriuretic peptide; CK-MB, creatine kinase-MB; LDH, lactate dehydrogenase; SBP, systolic blood pressure; DBP, diastolic blood pressure; MBP, mean blood pressure

Figure 2A–C present the statistical results of systolic blood pressure, diastolic blood pressure, and mean arterial pressure, respectively, of the two groups of rats at six time points in 24 h. The CM-group rats showed significantly higher systolic blood pressure [08:00 (p < 0.05), 12:00 (p < 0.05), 16:00 (p < 0.05), 20:00 (p < 0.001), and 24:00(p < 0.05)], diastolic blood pressure [08:00 (p < 0.05), 12:00 (p < 0.05), 16:00 (p < 0.05), 20:00 (p < 0.001) and 04:00 (p < 0.05)] and mean arterial pressure [08:00 (p < 0.05), 12:00 (p < 0.05), 16:00 (p < 0.05), and 20:00 (p < 0.001)] compared to the CA-group rats at all time points. The blood pressure values of the two groups of rats at the remaining time points were not statistically different.

The results of the blood pressure meta2d_pvalue, mata2d_AMP, and the meta2d_phase analysis for the CA and the CM groups of rats at the six time points during the 24-h LD cycle are shown in the Fig. 2D–F. The meta2d_pvalue of the systolic blood pressure and the mean arterial pressure for the CA rats was < 0.05, the meta2d_pvalue of the diastolic blood pressure was > 0.05 (Fig. 2D). But the meta2d_pvalue of the systolic blood pressure, diastolic blood pressure and mean arterial pressure for the CM rats was > 0.05 (Fig. 2D). The meta2d_AMP values of the systolic blood pressure, diastolic blood pressure, and the mean arterial pressure were lower in the CM rats compared to the CA rats (Fig. 2E). The meta2d_phase values of the systolic blood pressure were lower in the CM rats compared to the CA rats, but the meta2d_phase values of the diastolic blood pressure and the mean arterial pressure were higher in the CM rats compared to the CA rats (Fig. 2F).

### Weight analysis

Figure 2G and H show the results of the weight analysis performed at 08:00 after and before modeling. The results showed that there were no statistically significant differences in body weight between the two groups of rats before and after modeling (*p* > 0.05).

### Serological testing

Figure 2I, J, and K, respectively, present the levels of LDH, BNP, and CK-MB in the serum of the two groups of rats, revealing that the differences were not statistically significant (*p* > 0.05). Figures 2L and M present the levels of ANGII in the two groups measured at 08:00 and 20:00 after modeling, respectively. Figure 2L shows that at 08:00, the levels of ANGII were significantly higher in the CM group versus the CA group (*p* < 0.01). Figure 2M shows that at 20:00, the average levels of ANGII were higher in the CM group versus the CA group; however, the difference was not statistically significant (*p* = 0.222).

### Heart ultrasound

**Fig. 3:**
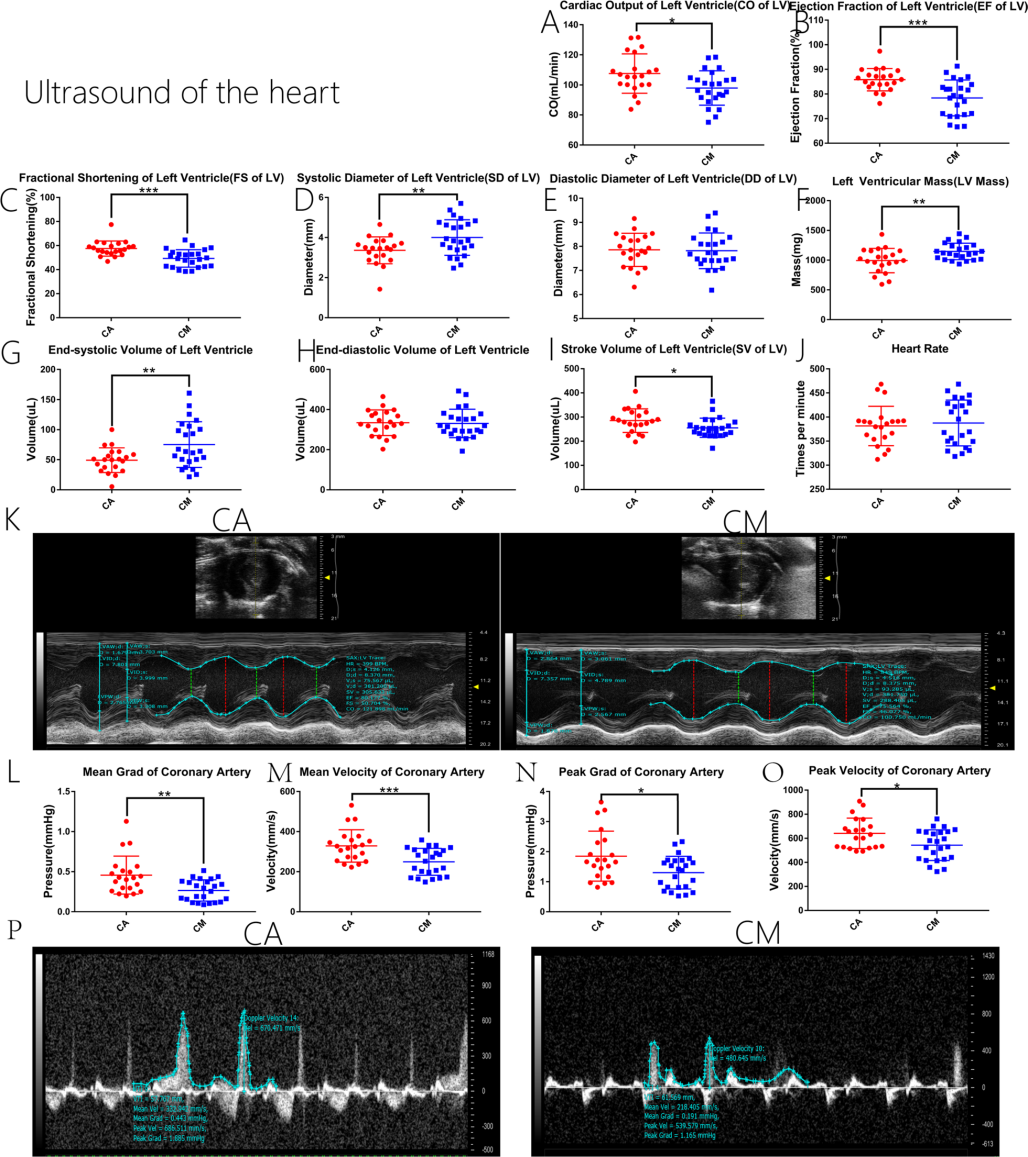
**A–J** Measurement results of M-Mode. **A–J** present the results for cardiac output per minute (CO), ejection fraction (EF), short axis shortening rate (FS), systolic diameter (SD), diastolic diameter (DD), left ventricular weight (LV Mass), end-systolic volume, end-diastolic volume, stroke volume (SV), and heart rate (HR), respectively. **K** M-mode measurement images and analysis results for the two groups of rats. **L–O** Mean transvalvular pressure, mean velocity, peak transvalvular pressure, and peak velocity of coronary blood flow in the two groups of rats. **P** Blood flow chart and analysis results for the coronary arteries of the two groups of rats measured using color Doppler. *p < 0.05, **p < 0.01, ***p < 0.001 (two-sample *t*-test)

Figure 3A–C and I present the results for left ventricular (LV) cardiac output per minute (*p* < 0.05), LV ejection fraction (*p* < 0.001), LV short axis shortening rate (*p* < 0.001), and LV stroke output per minute (*p* < 0.05), respectively. The findings showed that these indicators of heart function were significantly reduced in CM rats versus CA rats.

Figure 3D, F, and G present the results for the LV systolic diameter (*p* < 0.01), LV weight (*p* < 0.01), and LV end-systolic volume (*p* < 0.01), respectively. The data show that the three indicators were significantly higher in CM group versus the CA group.

Figures 3E, H, and J present the results for the LV diastolic diameter, LV end diastolic volume, and heart rate, respectively. The evidence did not show statistically significant differences (*p* > 0.05) in these indicators.

Figures 3L–O illustrate the results for the mean transvalvular pressure, mean flow velocity, peak transvalvular pressure, and peak flow velocity of the coronary arteries of the heart, respectively. The data show that the mean transvalvular pressure (*p* < 0.01), mean flow velocity (*p* < 0.001), peak transvalvular pressure (*p* < 0.05), and peak flow velocity of the coronary arteries (*p* < 0.05) were significantly reduced in the CM group compared with the CA group.

### Analysis of heart sections by Oil Red O staining

**Fig. 4:**
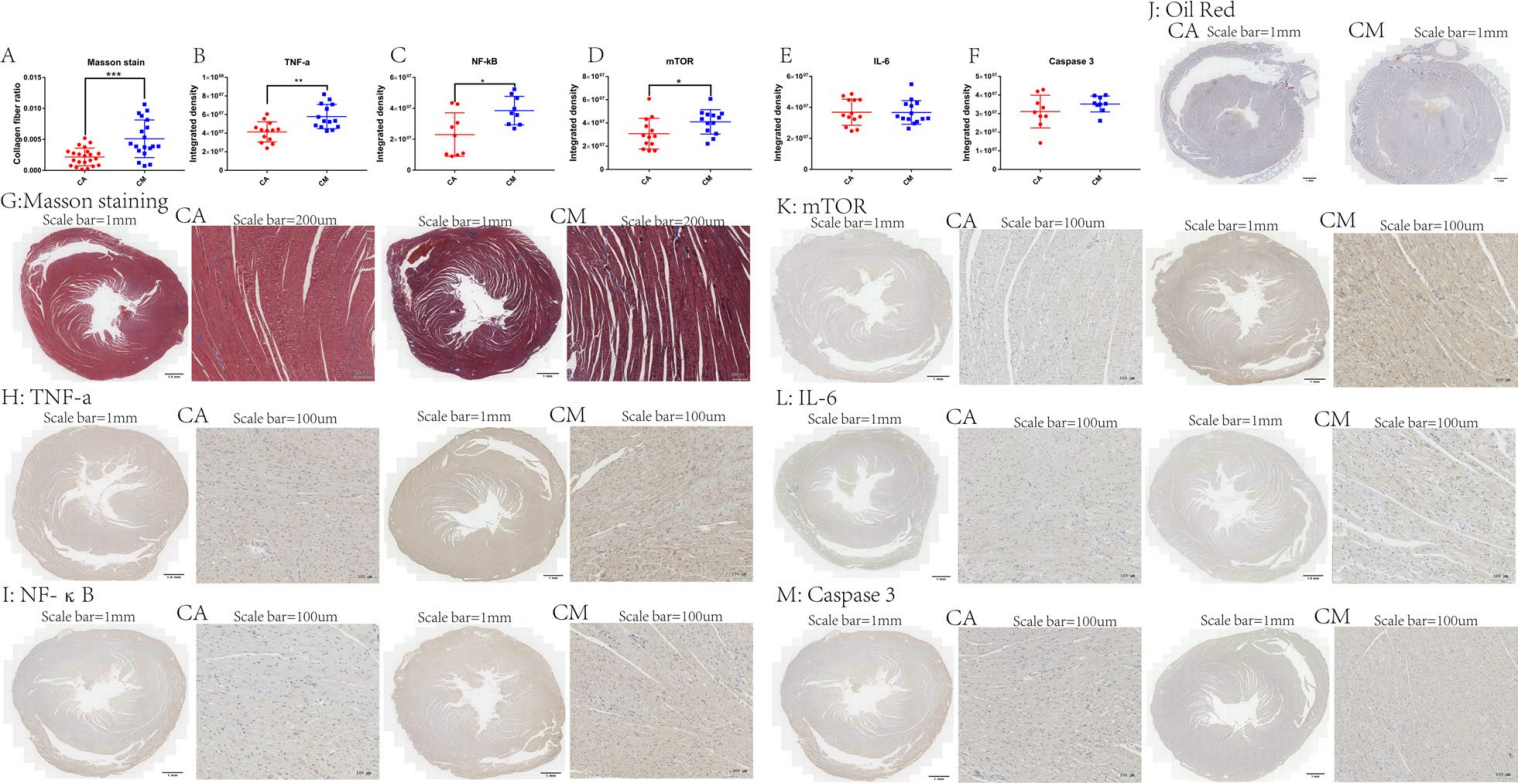
**A** Ratio of collagen fibers in heart sections obtained from the two groups of rats, measured by Masson’s trichrome staining. Levels of TNF-a (**B**), NF-κB (**C**), mTOR (**D**), IL-6 (**E**), and CASP3 (**F**) measured by immunohistochemical analysis of heart sections. *p < 0.05, **p < 0.01, ***p < 0.001 (two-sample *t*-test). **G** Masson’s trichrome staining of sections obtained from the two groups of rats. Immunohistochemical analysis of sections stained for TNF-a (**H**), NF-κB (**I**), mTOR (**K**), IL-6 (**L**), and CASP3 (**M**). Yellow–brown represents positive expression. **J** Staining with Oil Red O (scale: 1 mm). Abbreviations: CASP3, caspase 3; IL-6, interleukin-6; mTOR, mammalian target of rapamycin; NF-κB, nuclear factor-kappa B; TNF-a, tumor necrosis factor-alpha

Staining with Oil Red O did not reveal changes in lipid droplets or myocardial fat in the heart sections obtained from the two groups of rats (data not shown). Figure 4A presents the results of Masson’s trichrome staining, showing that the content of collagen fibers in the heart was significantly increased in CM rats compared with CA rats (*p* < 0.001).

### Immunohistochemistry

Figure 4B, C, and D demonstrate the results of the immunohistochemical analysis for TNF-α (*p* < 0.01), NF-κB (*p* < 0.05), and mTOR (*p* < 0.05), respectively. The data show that the expression of these proteins was significantly increased in CM rats versus CA rats.

Figure 4E and F present the results of the immunohistochemical analysis for IL-6 and CASP3, respectively. The evidence shows that the difference in the expression of these two proteins between CM rats and CA rats was not statistically significant (*p* > 0.05).

### TEM

**Fig. 5:**
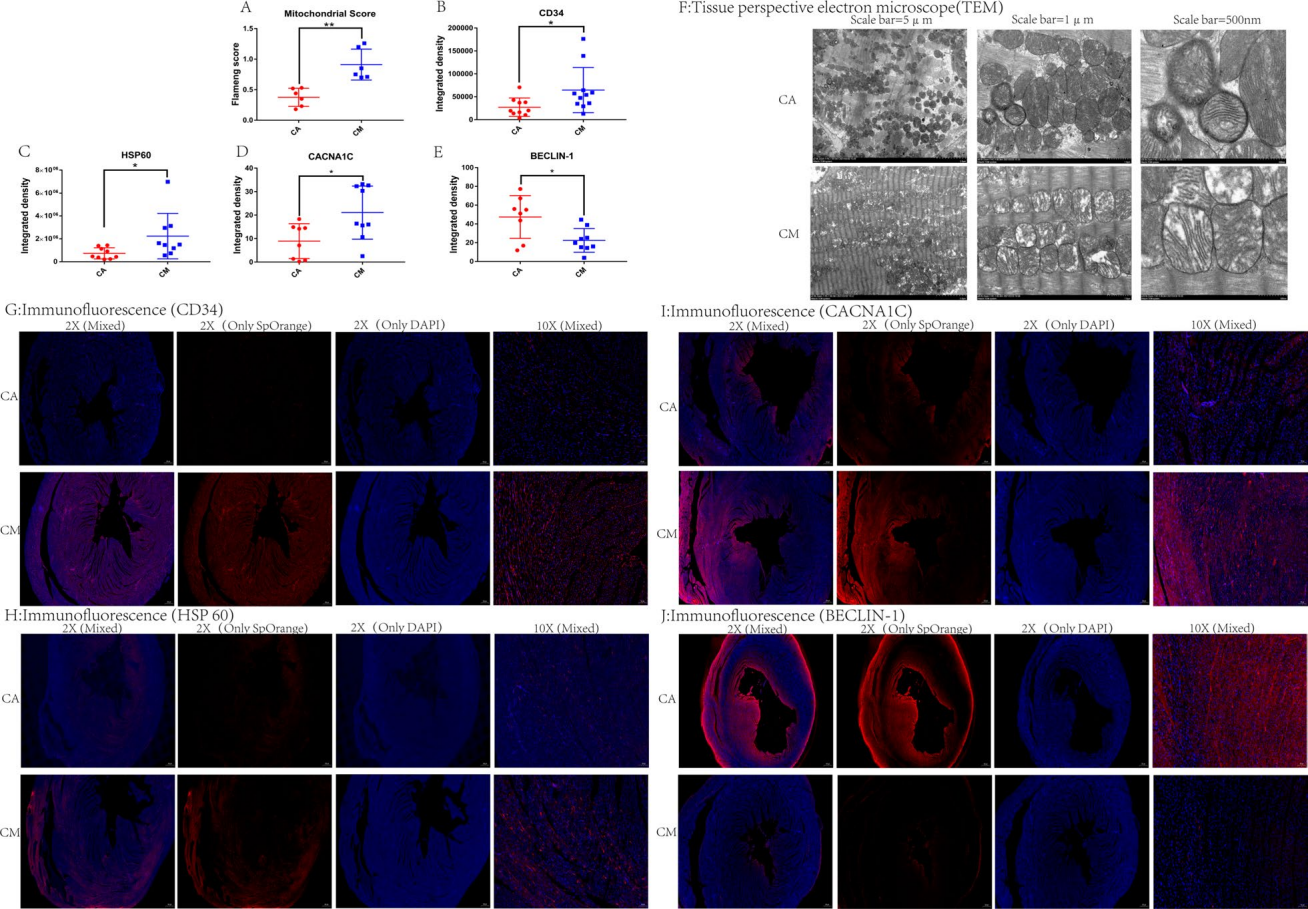
**A** The Flameng score of myocardial mitochondria measured using a fluoroscopy electron fiberscope. Expression of CD34 (**B**), HSP60 (**C**), CACNA1C (**D**) and BECLIN-1 (**E**) in the two groups of rats measured by immunofluorescence staining. *p < 0.05, **p < 0.01, ***p < 0.001 (two-sample *t*-test). (**F**) Transmission electron microscope scanning images of the myocardial mitochondria from the two groups of rats. A large number of myocardial mitochondria in the CM group showed vacuolar changes and cristae fractures, indicating a significant increase in ruptured mitochondria. Immunofluorescence staining for CD34 (**G**), HSP60 (**H**), CACNA1C (**I**) and BECLIN-1 (**J**). Blue represents the nucleus stained with DAPI, and red represents positive expression. Abbreviations: DAPI, 4′,6-diamidino-2-phenylindole; HSP60, heat shock protein 60; CACNA1C, Calcium Voltage-Gated Channel Subunit Alpha1 C; BECLIN-1, Coiled-Coil, Moesin-Like BCL2-Interacting Protein

Figure 5A presents the Flameng score of myocardial mitochondria in the two groups of rats. The results show that the Flameng score was significantly higher in CM rats versus CA rats (*p* < 0.01).

### Immunofluorescence

Figure 5B-E present the results of the immunofluorescence analysis for the levels of the myocardial CD34, HSP60, CACNA1C and BECLIN-1, respectively. Compared with rats in CA group, the expressions of CD34 (*p* < 0.05), HSP60 (*p* < 0.05) and CACNA1C (*p* < 0.05) in CM rats were significantly increased, and the expression of BECLIN-1 was significantly decreased (*p* < 0.05).

### WB of myocardial tissue

**Fig. 6:**
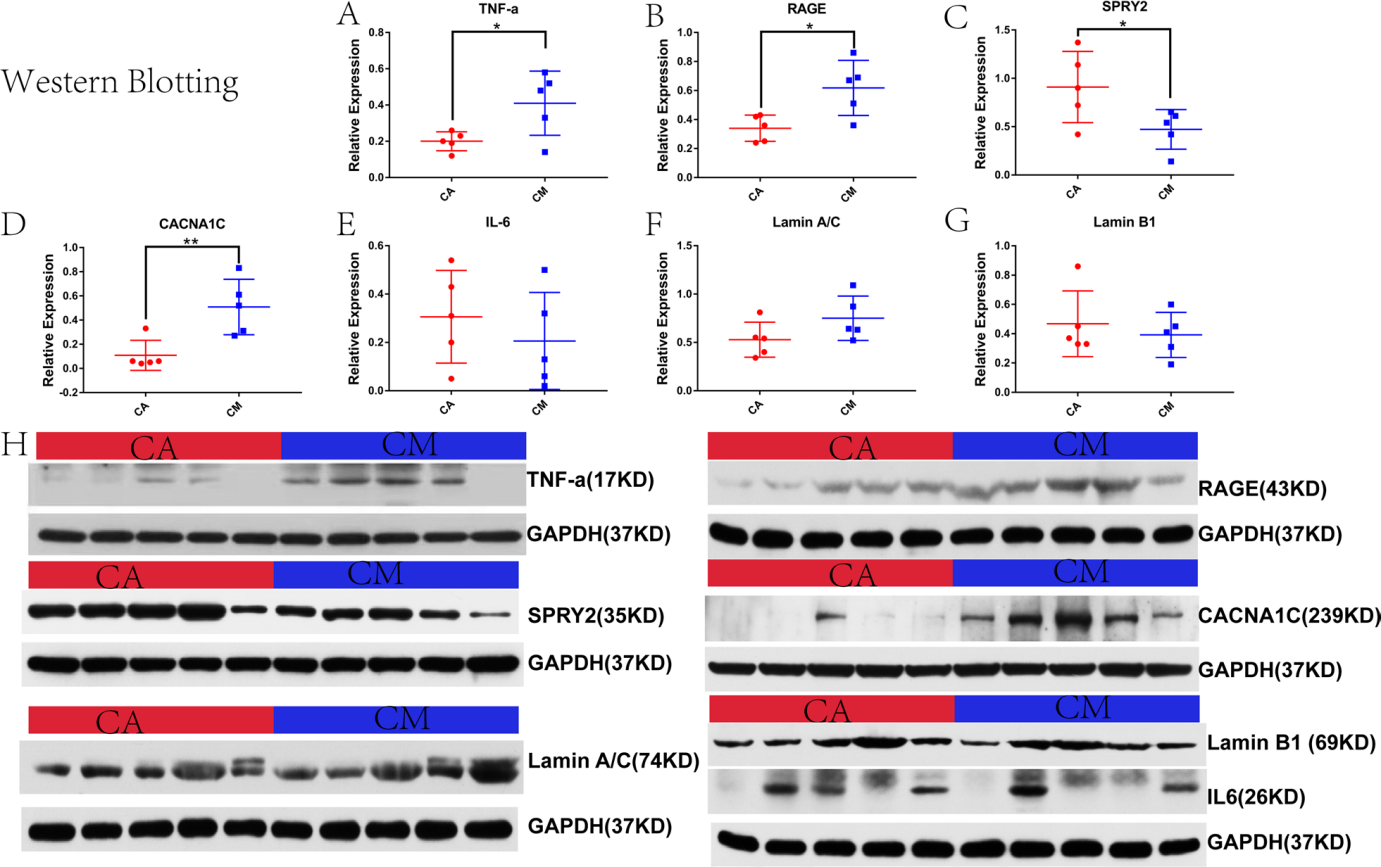
Relative expression levels of TNF-a (**A**), RAGE (**B**), SPRY2 (**C**), CACNA1C (**D**), IL-6 (**E**), lamin A/C (**F**), and lamin B1 (**G**) in the apical tissues of rats from the two groups, measured by western blotting. *p < 0.05, **p < 0.01, ***p < 0.001 (two-sample *t*-test). **H** Bands of the above proteins. Abbreviations: IL-6, interleukin-6; RAGE, receptor for advanced glycosylation end-product specific; SPRY2, sprouty RTK signaling antagonist 2; TNF-a, tumor necrosis factor-alpha; CACNA1C, Calcium Voltage-Gated Channel Subunit Alpha1 C

Figure 6A–D present the levels of the myocardial TNF-α, RAGE, SPRY2 and CACNA1C proteins in the two groups of rats. The data show that compared with the CA group, the relative expression of TNF-α (*p* < 0.05), RAGE (*p* < 0.05) and CACNA1C (*p* < 0.01) were significantly increased in the CM group, whereas that of SPRY2 was significantly decreased (*p* < 0.05).

Figure 6E–G illustrate the relative expression of IL-6, lamin A/C, and lamin B1 in the two groups of rats, respectively. The evidence reveals that differences in the relative expression of these proteins in CM rats and CA rats were not statistically significant (*p* > 0.05).

### The statistical results of serological substances and qPCR of myocardial tissue at six time points in 24 h.

**Fig. 7:**
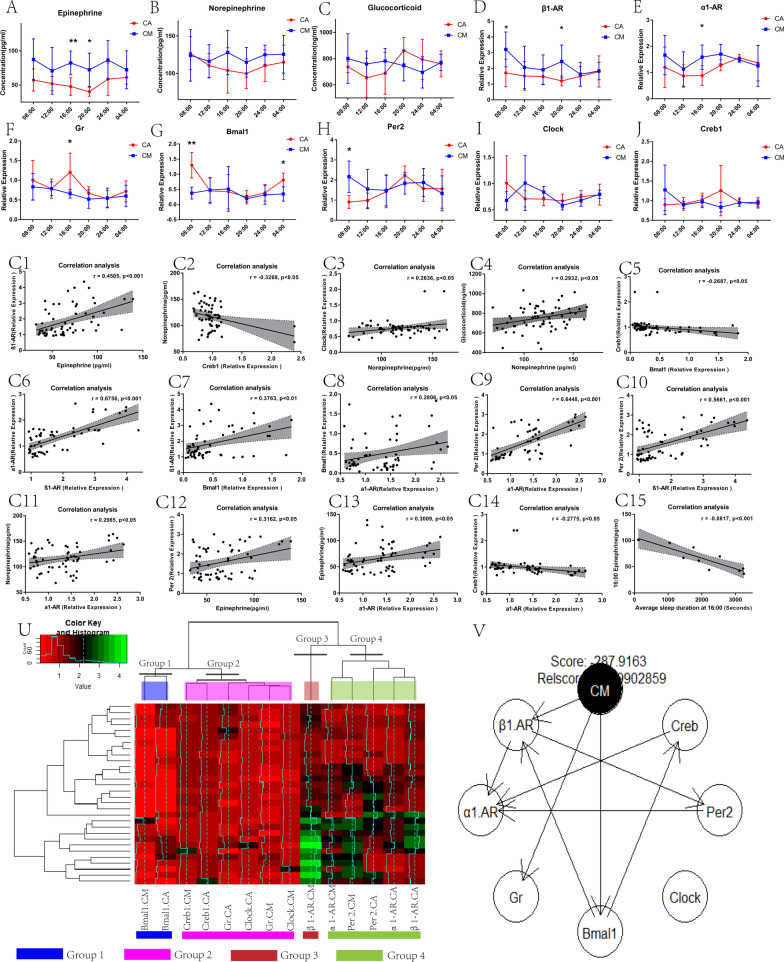
**A-C** represent the statistical results of the concentration of serum EPI, NE, and COR, respectively, in the two groups of rats at six time points in 24 h. **D**–**J** represent the relative expression of the myocardial *β1-AR*, *α1-AR*, *Gr*, *Bmal1*, *Per2*, *Clock*, *and Creb1* respectively, in the two groups of rats at six time points in 24 h. The two independent samples *t*-test was used to compare the two groups at each time point. *p < 0.05, **p < 0.01, ***p < 0.001. (**C1-C15**) represent the correlation analysis between the two factors, respectively. The U represents a two-way clustering heat map of gene expression in myocardial tissue, which is classified using a hierarchical clustering method. Red represents low relative expression, and green represents high relative expression. The V represents the Bayesian network diagram of the interaction between CM and myocardial genes calculated using the heuristic mode. When there is a line between two factors, it indicates that the two factors have a causal relationship (Parents-Descendants), the arrow points to the result (Descendants), and the other end of it is the cause (Parents)

Figure 7A-C present the statistical results of serum EPI, NE, and COR, respectively, of the two groups of rats at six time points in 24 h. Compared with the CA-group rats, the CM-group rats had significantly higher serum EPI levels at 16:00 (*p* < 0.01) and 20:00 (*p* < 0.05). There were no statistically significant differences in the NE (*p* > 0.05) and COR (*p* > 0.05) levels between the two groups of rats at six time points in 24 h. However, it can be seen from the statistical graph that the COR of the two groups of rats showed an opposite trend.

Figure 7D–J represent statistical results of two groups of rats at six time points myocardium for *β1-AR*, *α1-AR*, *Gr*, *Bmal1*, *Per2*, *Clock*, and *Creb1* gene expression, respectively. The statistical results show that the expression of the *β1-AR* gene in CM group rats was significantly increased at 08:00 (*p* < 0.05) and 20:00 (*p* < 0.05) compared to CA group rats. The expression of *α1-AR* was significantly increased at 16:00 (*p* < 0.05). The expression of the *Gr* gene was significantly decreased at 16:00 (*p* < 0.05). The expression of *Bmal1* was significantly decreased at 04:00 (*p* < 0.05) and 08:00 (*p* < 0.01). The expression of *Per2* was significantly increased at 08:00 (*p* < 0.05). The remaining genes showed no significant statistical differences between the groups in the remaining time points.

Figure 7C1–C14 show statistically significant correlations between EPI and *β1-AR* (*r* = 0.4505, *p* < 0.001, *R*^*2*^ = 0.2029), NE and *Creb1* (*r* = − 0.3268, *p* < 0.05, *R*^*2*^ = 0.1068), NE and *Clock* (*r* = 0.2636, *p* < 0.05, *R*^*2*^ = 0.06948), NE and COR (*r* = 0.2932, *p* < 0.05, *R*^*2*^ = 0.08595), Bmal1 and *Creb1* (*r* = − 0.2687, *p* < 0.05, *R*^*2*^ = 0.07218), *α1-AR* and *β1-AR*(*r* = 0.6756, *p* < 0.001, *R*^*2*^ = 0.4565), *Bmal1* and *β1-AR* (*r* = 0.3763, *p* < 0.01, *R*^*2*^ = 0.1416), *Bmal1* and *α1-AR* (*r* = 0.2806, *p* < 0.05, *R*^*2*^ = 0.07871), *Per2* and *α1-AR* (*r* = 0.6448, *p* < 0.001, *R*^*2*^ = 0.4157), *Per2* and *β1-AR* (*r* = 0.5661, *p* < 0.001, *R*^*2*^ = 0.3205), *α1-AR* and NE (*r* = 0.2965, *p* < 0.05, *R*^*2*^ = 0.08791), EPI and *Per2* (*r* = 0.3162, *p* < 0.05, *R*^*2*^ = 0.09998), EPI and *α1-AR* (*r* = 0.3009, *p* < 0.05, *R*^*2*^ = 0.09057), and *α1-AR* and *Creb1* (*r* = − 0.2775, *p* < 0.05, *R*^*2*^ = 0.07701), respectively.

Figure 7C15 shows that the average sleep duration at 16:00 is negatively correlated with EPI (*r* = -0.8817, *p* < 0.001, *R*^*2*^ = 0.7775).

Figure 7U, through a hierarchical clustering method performed by drawing a two-way heat map of clustering analysis, shows grouping of the expression levels of the above genes measured in myocardial tissue. The results show that after the genes were classified, the following four groups can be formed. Group 1: *Bmal1.CM, Bmal1.CA*; Group 2: *Creb1.CM, Creb1.CA, Gr.CA, Clock.CA, Gr.CM, Clock.CM*; Group 3: *β1-AR.CM*; and Group 4: *α1-AR.CM, Per2.CM, Per2.CA, α1-AR.CA, β1-AR.CA.*

Figure 7V shows a Bayesian network diagram demonstrating that CM, as a cause (Parents), affects the expression of the following genes (Descendants) through the following influence pathways. 1. CM-*(β1-AR)-(α1-AR)*; 2. CM-*(β1-AR)-Per2-(α1-AR)*; 3. CM-*Gr*; 4. CM-*Bmal1-(β1-AR)- (α1-AR)*; 5. CM-*Bmal1-(β1-AR)-Per2-(α1-AR)*; and 6. CM- *Bmal1-Creb1-(α1-AR)*.

## Discussion

After 3 months of modeling, the Clocklab behavior chart and HomeCage sleep chart showed that the CA and CM groups showed great differences in behavioral rhythms. The rats in the CA group were in the resting phase and sleep-concentration stage from 08:00 to 20:00 and were in the active phase and awakening-concentration stage from 20:00 to 08:00 the next day. However, the activity/rest rhythm of the rats in the CM group changed significantly. Compared with the rats in the CA group, the rats in the CM group showed a decrease in the rhythm amplitude and a prolonged rhythm period. At the same time, CM also changed the sleep/wake cycle of the rats, which manifested as a prolonged sleep cycle and decentralized sleep. The findings show that at 16:00, under the normal photoperiod, the sleep duration of CM rats was significantly reduced, with a short awakening period (Fig. 1F). The obvious differences in behavior between the two groups of rats indicate that this modeling was successful.

This study demonstrated significant changes in the heart structure and function of the adolescent CM group rats. The hearts of CM rats demonstrated significantly higher myocardial collagen fiber content and increased disintegration of myocardial mitochondria. The structural changes in the heart are associated with significant alterations in the cardiac contraction and pumping functions. The cardiac ultrasound results demonstrated significant reduction in the ejection fraction, stroke volume, short axis shortening rate, and the coronary blood flow rate in the hearts of the CM group rats. Furthermore, the hearts of the CM group rats showed increased systolic diameter and systolic volume, thereby suggesting reduced heart pumping action and the self-supply function of the coronary artery. The reduced heart pumping function can cause insufficient systemic circulation and pose a great threat to the health of adolescent individuals that are experiencing CM. Therefore, these results support that CM should generate concern. According to these research results, CM causes heart damage in CM rats through the following 5 factors:

1. CM increases the preload of the heart by increasing the serum EPI content and the expression of myocardial β1-AR and α1-AR genes.
  This study confirmed that the serum EPI and NE contents of the CA group were at lower levels during the day, while the serum EPI and NE concentrations were elevated at night. Correspondingly, gene expression of *α1-AR* and *β1-AR* in the myocardium of rats in the CA group showed similar rhythms. These physiological characteristics set the functional state of the heart in a rhythm of work/rest switching [19], such that after the heart works normally, it can obtain sufficient rest by slowing down the heart rate and reducing the contractility of the myocardium [20, 21]. However, as seen in Fig. 7A, D, and E, the serum EPI concentrations in CM rats and the expression of myocardial *β1-AR* and *α1-AR* genes were higher than those in CA rats. This indicates that CM significantly increased the EPI levels in rats and significantly enhanced the positive effect of EPI on the myocardium. Therefore, CM will inevitably lead to an increased heart rate and myocardial contractility through the above-mentioned mechanisms [22, 23]. In particular, the results of this experiment show that both the EPI concentration and *β1-AR* gene expression of CM rats were continuously elevated over 24 h, which could lead to excessive fatigue of the myocardium and could cause severe damage to the myocardium long-term [24, 25]. These results also explain why many CM people generally feel uncomfortable symptoms such as palpitations or chest tightness after staying up late for work. [26] As to the reason for the abnormal increase in EPI caused by CM, it is likely that it is closely related to the abnormal rhythm of activity [26]. Correlation analysis showed that the sleep time of rats at 16:00 was negatively correlated with serum EPI, and the correlation was statistically significant, which indicates that the sleep quality of rats in the resting phase affected the serum EPI content through the sympathetic nervous system [27].
  Previous studies have shown that long-term shift work increased the blood pressure and reduced the urinary epinephrine levels [3]. This was not consistent with the physiological changes associated with increased blood epinephrine levels. However, CM also increased the excitability of the sympathetic nerves and reduced the excitability of the vagus nerve [3]. The enhanced activity of the sympathetic nervous system promotes contraction of the renal afferent arterioles and reduces the effective filtration rate of the kidneys, thereby significantly decreasing the excretion of urinary epinephrine. Furthermore, in the animal experiments [28], CM reduced the amount of urine and excretion of the sodium ions by promoting the reabsorption of water and salt by the kidneys through the RAAS system. These data suggested that excitation of the sympathetic nerves by CM increased the serum levels of EPI and decreased the levels of urine EPI. Our data also demonstrated that CM increased the levels of serum EPI and Ang-II in the rats. This suggested that CM increased the sympathetic nerve activity in the adolescent rats and was consistent with previously reported findings.
2. CM increases AngII and blood pressure, and disrupts the circadian rhythm of blood pressure, leading to an increase in cardiac afterload.
  CM significantly increased the blood pressure of rats at multiple time points during the 24-h cycle, especially during the resting phase (daytime). This increased the cardiac afterload. Furthermore, elevated blood pressure in the CM rats during the daytime may be related to shortened sleep. Our study also demonstrated that CM altered the blood pressure rhythm. The alterations in the systolic blood pressure and the mean arterial pressure rhythm demonstrated that CM disrupted the circadian rhythm of blood pressure in the adolescent rats. CM also decreased the rhythmic amplitudes of the systolic, diastolic, and the mean arterial pressures, and altered the blood pressure phase in the adolescent rats. Because CM simultaneously reduced the amplitudes of the behavioral and the blood pressure rhythms in the adolescent rats, we postulated that the alterations in the behavioral rhythm contributed to the disruption in the blood pressure rhythm. In the CM rats, the serum ANG-II levels were significantly higher at 08:00; serum EPI levels were significantly increased at 16:00 and 20:00; and myocardial *β1-AR* levels were significantly higher at 08:00 and 20:00. These molecular indicators confirmed the changes in the blood pressure rhythm during the resting phase in the adolescent CM rats.
  The systolic blood pressure and the mean blood pressure of the CA rats demonstrated circadian rhythm. The blood pressure values of the CA rats were reduced during the day (resting phase) and increased at night (active phase). This allowed sufficient recovery time for the heart. However, circadian rhythm was not observed for the blood pressure in the CM rats. They demonstrated significantly higher blood pressure during the day (rest phase) and at night (activity phase). This significantly increased the afterload for longer periods of time and shortened the rest time for the heart, thereby causing myocardial damage [29].
  On one hand, increased cardiac load will cause the relative blood and oxygen supplies of the myocardium to be insufficient [30, 31]. The results of the cardiac ultrasonography show that the slowing of the coronary blood-flow rate will directly lead to the reduced functional myocardial blood supply. The increased expression of CD34 indicates that the myocardium forms more collateral circulation indicating that the myocardium of CM rats is in a relatively ischemic and hypoxic state [32]. On the other hand, the increase in the preload and afterload of the heart will inevitably increase the workload of the mitochondria, which will eventually lead to an increase in mitochondrial disintegration [31, 33]. In addition, studies have confirmed that increased secretion of serum EPI can directly cause increased mitochondrial destruction [34]. In this experiment, the increased HSP60 concentrations in the myocardium of CM rats and the increased disintegration of mitochondria as shown by transmission electron microscopy are the undesirable consequences of these factors [35–37]. Furthermore, CM increased the expression levels of mTOR and decreased the expression levels of BECLIN-1, thereby suggesting suppression of mitochondrial autophagy in the myocardial tissues [38, 39]. Especially with the increase of mitochondrial dysfunction, the decline of autophagy indicates that the ability of the myocardium to clear away damaged mitochondria is weakened [40]. This series of undesirable changes will lead to further enhancement of the secondary damage of CM to the myocardium.
3. CM can negatively affect heart rate through molecular pathways.
  Western blotting analysis demonstrated that the expression levels of SPRY2 were significantly reduced in the myocardium of the CM rats. SPRY2 regulates a normal heart rate by modulating the activity of the calcium ion channels in the myocardium and the sinoatrial node; therefore, reduced SPRY2 is associated with arrhythmias [41–43]. The L-type calcium channels participate in the formation and propagation of the action potential during the rapid depolarization (plateau) phase of the myocardium [44, 45]. Our data demonstrated that the expression levels of CACNA1C were significantly increased and SPRY2 expression levels were significantly reduced in the myocardium of the CM rats. This suggested that CM significantly increased the susceptibility of arrhythmia.
4. CM causes abnormal glucocorticoid rhythms and reduces myocardial Gr gene expression, triggering a myocardial inflammatory response.

Serological results showed that the 24-h rhythm of COR in CM rats showed an opposite trend to that of CA rats. At the genetic level, there were more obvious differences. First, the expression of the *Gr* gene in the myocardium of CM rats was significantly lower than that of CA rats at ZT8 (16:00). Second, the rhythm of *Gr* gene expression in CM rats and CA rats also showed different trends. This shows that CM not only reduces the expression of *Gr*, but also affects the rhythm of the glucocorticoid pathway. Glucocorticoids and their receptors can produce a strong anti-inflammatory effect and, to a certain extent, play a protective effect on the myocardium [46]. Its weakened effect can lead to an enhanced inflammatory response. The results from the immunohistochemistry and WB studies show that CM can increase myocardial TNF-α and RAGE-NF-κB by reducing the expression of *Gr*, [47] triggering an enhanced inflammatory response, and causing serious damage to the heart [48, 49].

5. The changes described above are highly correlated with the abnormal rhythmic expression of rat heart clock genes caused by CM. Several statistical results suggest that CM possibly regulates the function and rhythm of the heart by affecting clock genes and their molecular pathways.

Figure 7 shows that the expression of *Clock* and *Bmal1* decreased in CA rats under light, and the expression increased in the dark, while *Per2* showed the opposite trend. However, the statistical results showed that the expression of *Bmal1* in CM rats was significantly lower than the expression in CA rats at 04:00 and 08:00, indicating that CM blocked the expression of the *Bmal1* gene in rat myocardium in the dark, or accelerated its decomposition. In addition, CM also caused the 24-h rhythmic changes of the *Bmal1* and *Clock* genes in the rat heart to show different or even opposite trends from those of the CA group. In the study of *Per2* gene at 08:00, in CM rats, it was significantly higher than that of CA rats. These results indicate that CM triggers the endogenous rhythm disorder of the rat heart from the level of clock genes [50].

This study suggested that CM altered the expression of the functional receptor genes such as *α1-AR*, *β1-AR*, and *Gr*, possibly by disrupting the expression levels of the *Bmal1*, *Creb1,* and *Per2* genes, which regulate the molecular oscillation circuit of the clock genes. Bayesian network results showed that the decreased levels of *Bmal1* directly affects the expression levels of *β1-AR* and *Creb1*. The expression of *α1-AR* is regulated both directly and indirectly by *β1-AR* via *Per2*. *Creb1* is not a clock gene, but plays an intermediary role in the regulation of biological rhythms by modulating the expression levels of various clock genes involved in the molecular oscillation circuit. Furthermore, *Creb1* plays an important role in the heart contractile function and energy metabolism by modulating the activity of the cardiac ion channels through oxidative phosphorylation [51–53]. This study demonstrated that *Creb1* regulated the expression levels of *α1-AR*. This suggested a significant role for the clock genes involved in the molecular oscillation circuit in the regulation of the functional adrenergic receptors. These facts all indicate that the change of the clock gene oscillation circuit caused by CM not only causes the disturbance of the cardiac rhythm, but also has an important adverse effect on the functional receptors of the heart through the disturbed clock gene oscillation circuit.

Bray et al. [54] demonstrated that heart rate was significantly reduced in the cardiomyocyte-specific circadian clock mutant mice. Schroder et al. [55] reported dilated cardiomyopathy and slow heart rate in the *Bmal1* knockout mice. Schroeder et al. [55] also demonstrated that knockout of the endogenous clock genes increased the risk of cardiac arrhythmia. The researchers [55] found that the physiological function and rhythm of the heart is regulated by the neuro-humoral regulatory mode, which relies on the regulation of the central nervous system, and the clock gene molecular oscillatory circuit, which relies on the regulation of the endogenous clock genes. Furthermore, the exogenous neuro-humoral regulatory mode plays a major regulatory role, whereas the endogenous clock genes play a secondary regulatory role [55]. In our experiments, we used IP for generating the CM model rats. Finally, we observed significant elevation of serum EPI and ANGII levels in the CM rats were associated with changes in the cardiac biological rhythm. This indicated that CM altered the central “nerve-humoral” regulation and was manifested by significant changes in the blood pressure and serum EPI levels. In addition, CM induced changes in the expression levels of the endogenous clock genes, *Bmal1* and *Per2*, in the heart. This suggested that CM altered both the central “neuro-humoral” regulatory pathways and the endogenous clock genes, thereby affecting the heart rhythm and functions. These effects of CM on the heart are more complex than the effects of the clock gene knockout alone. Therefore, our study suggested that the negative effects of CM on the heart were caused mostly by the alterations of the "neuro-humoral" regulatory pathway.

Fortunately, although CM caused a decrease in cardiac function in the adolescent rats, serum BNP, CK-mB, and LDH in CM rats did not significantly increase. There was also no significant difference in the expression of myocardial Caspase-3. This shows that in the three-month modeling protocol, despite the increased destruction of myocardial mitochondria, decreased contractile function, and other adverse changes, CM had not yet caused a large-scale death of the myocardium. With the current CM phenomenon becoming more and more common, timely effective measures can limit or reverse myocardial damage. According to the results of this experiment, two feasible prevention or intervention programs might be effective. (1) The Bayesian network diagram (Fig. 7V) shows that CM can regulate multiple molecular pathways by affecting the myocardial *β1-AR* gene. In addition to the direct effect of *β1-AR* on *α1-AR*, CM can affect the expression of *Per2* by affecting the expression of *β1-AR* (CM-(*β1-AR*)-*Per2*). This shows that *β1-AR* gene expression affects *Per2* gene expression, thus resetting the heart rhythm. In addition, the heat map results of gene expression (Fig. 7U) show that in the expression classification of all myocardial genes, the expression of the *β1-AR* gene in CM rats is a separate group, indicating that the change in expression of the *β1-AR* gene caused by CM is the most significant of all genes. Finally, combined with the increase in serum EPI content of CM rats, it indicates that the mechanism of CM's influence on the heart is different from common heart diseases such as coronary heart disease and viral myocarditis. The main harm lies in myocardial fatigue and endogenous cardiac rhythm disorders caused by the increased expression of adrenaline and its receptors due to CM. Therefore, interventional drugs for myocardial β receptors, such as beta blockers, can theoretically have an important protective effect on the heart [56]. (2) The decrease in sleep duration of CM rats at 16:00 indicates that CM caused changes in the sleep/wake cycle of rats, and this change was associated with the increase in EPI. Perhaps it is possible to consider giving drugs or other interventions to improve sleep and reduce the level of serum EPI. Of course, the feasibility of these interventions need to be demonstrated by further studies.

This study has some limitations. (1) In our molecular pathway analysis, we found that CM can affect the expression of *RAGE* and *SPRY2*; however, we did not investigate the upstream and downstream molecules of this molecular pathway. This will be addressed in future research. (2) During the 3-month study period, CM exerted a negative effect on the structure of the heart, but did not cause large-scale death of cardiomyocytes. Assessing the effect of prolonging the modeling time on cardiomyocytes warrants further investigation. (3) Although the Bayesian network results suggested that CM affected the functional adrenergic receptors by interfering with the clock genes, direct evidence was not shown in this study. Therefore, future studies are required to confirm the regulatory effect of the clock genes on the functional adrenergic receptors in the CM model.

In summary, this study demonstrated that CM disrupted the cardiac rhythm of the adolescent Wistar rats and significantly altered the blood pressure and the cardiac contractile and pumping function. Our study demonstrated significant alterations in the cardiac structure and function of the adolescent CM rats, thereby suggesting increased risk of heart disease (see Additional file 2: Fig. S2).

## Supplementary Information

**Additional file 1: Figure S1.** (**A**) The time list of this experiment. (**B**) The photoperiod of the two groups of rats. (**C**) The measurement items and number distribution of the two parts rats

**Additional file 2: Figure S2.** Summary of the findings in this study

**Additional file 3: **Experimental methods.

## Data Availability

The datasets used and/or analysed during the current study are available from the corresponding author on reasonable request.

## Abbreviations

ANGII: Angiotensin II
α1-AR: Adrenergic receptor-α1
β1-AR: Adrenergic receptor-β1
BECLIN-1: Coiled-Coil, Moesin-Like BCL2-Interacting Protein
BNP: Brain natriuretic peptide
CA: Circadian alignment
CACNA1C: Calcium Voltage-Gated Channel Subunit Alpha1 C
CK-MB: Creatine kinase-MB
CM: Circadian misalignment
COR: Glucocorticoid
Creb1: CAMP-response element binding protein 1
DAPI: 2-(4-Amidinophenyl)-6-indolecarbamidine dihydrochloride
GAPDH: Glyceraldehyde 3-phosphate dehydrogenase
Elisa: Enzyme-linked immunosorbent assay
EPI: Epinephrine
Gr: Glucocorticoid receptor
HSP60: Heat shock protein 60
IL-6: Interleukin-6
ILD: Inverted light/dark
IP: Inverted photoperiod
LD: Light/dark
LDH: Lactate dehydrogenase
mTOR: Mammalian target of rapamycin
NF-κB: Nuclear factor-kappa B
NE: Norepinephrine
Per 2: Period circadian regulator 2
qPCR: Quantitative polymerase chain reaction
RAGE: Receptor for advanced glycosylation end-product specific
SCN: Suprachiasmatic nucleus
SPRY2: Sprouty RTK signaling antagonist 2
TEM: Transmission electron microscopy
TNF-α: Tumor necrosis factor- alpha
WB: Western blotting
ZT: Zeitgeber
