# Supplementary material for: Harm of circadian misalignment to the hearts of the adolescent wistar rats
Source: J Transl Med. 2022 Aug 6;20:352. doi: 10.1186/s12967-022-03546-w (PMC9356460; doi:10.1186/s12967-022-03546-w)

Supplementary Figure 1 (Figure S1):

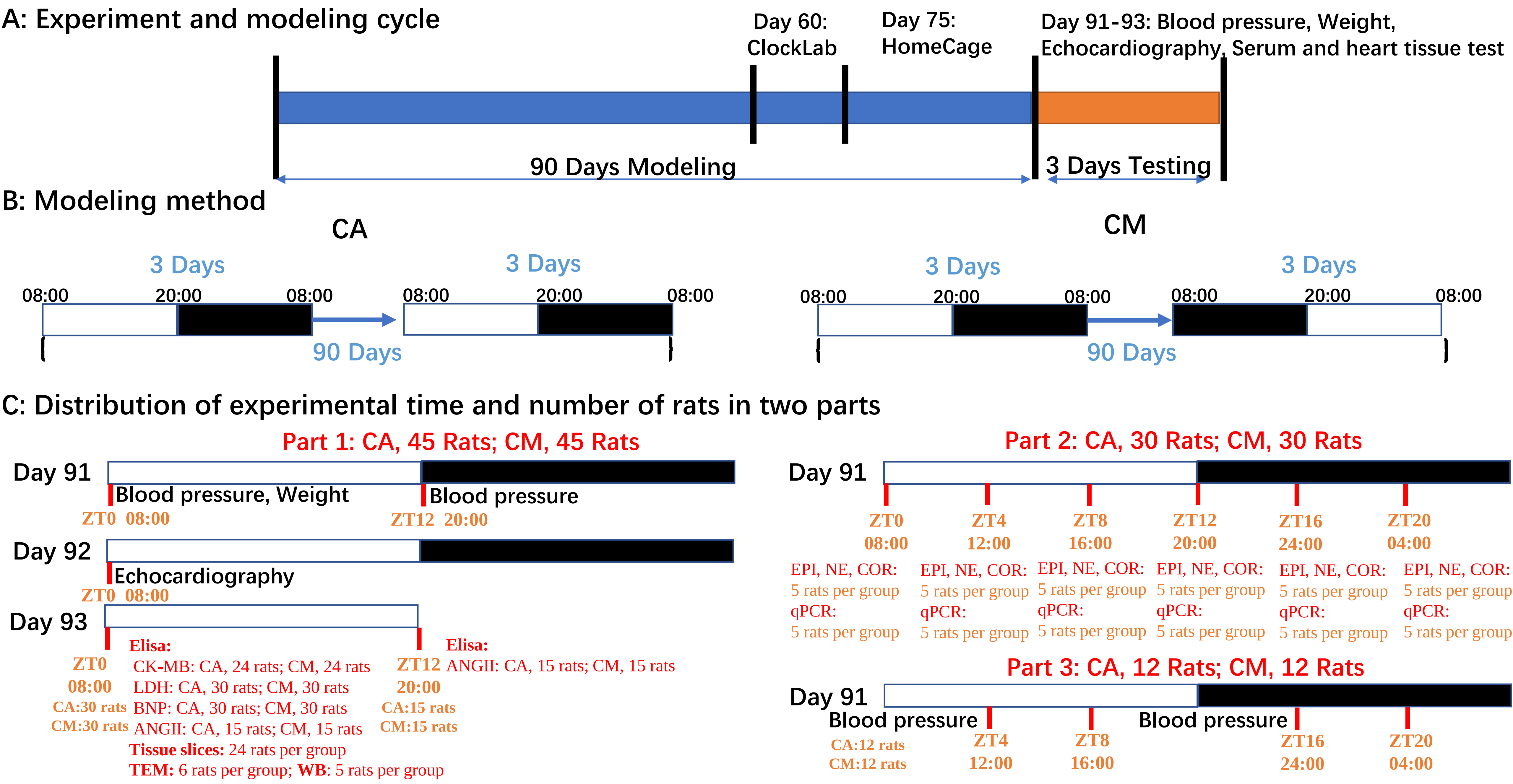

Supplement: Supplementary file 1 — Additional file 1: Figure S1. (A) The time list of this experiment. (B) The photoperiod of the two groups of rats. (C) The measurement items and number distribution of the two parts rats [file 12967_2022_3546_MOESM1_ESM.pdf]
