# Supplementary figures and images for: Harm of circadian misalignment to the hearts of the adolescent wistar rats
Source: J Transl Med. 2022 Aug 6;20:352. doi: 10.1186/s12967-022-03546-w (PMC9356460; doi:10.1186/s12967-022-03546-w)

Figure S2

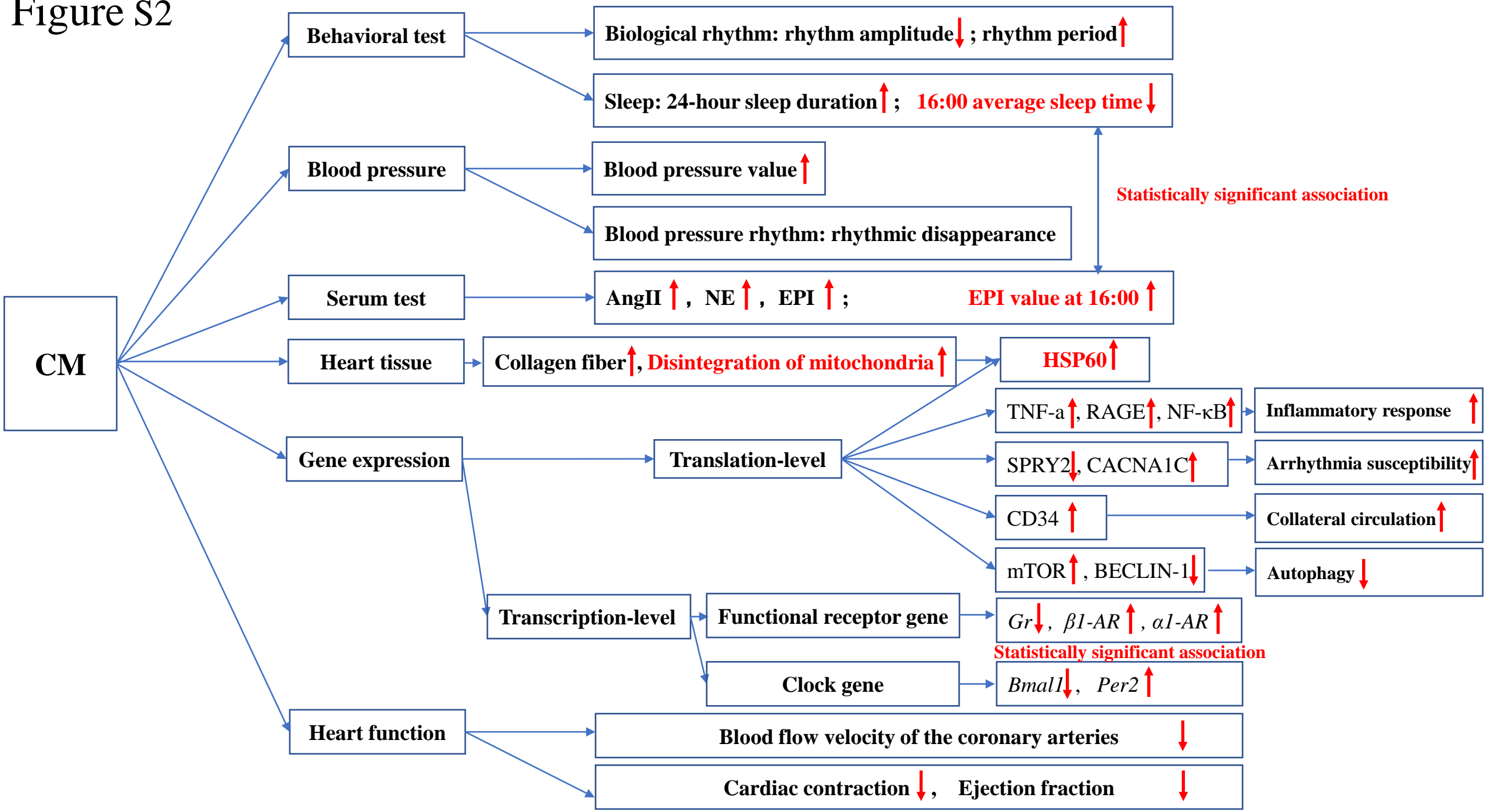

Supplement: Supplementary file 2 — Additional file 2: Figure S2. Summary of the findings in this study [file 12967_2022_3546_MOESM2_ESM.pdf]
