# Supplementary material for: Harm of circadian misalignment to the hearts of the adolescent wistar rats
Source: J Transl Med. 2022 Aug 6;20:352. doi: 10.1186/s12967-022-03546-w (PMC9356460; doi:10.1186/s12967-022-03546-w)
Supplement: Supplementary file 3 — Additional file 3: Experimental methods. [file 12967_2022_3546_MOESM3_ESM.docx]

**Supplementary Experimental Methods**

**Method of blood pressure measurement:** Blood pressure was measured using a six-channel rat non-invasive CODA sphygmomanometer (KENT, USA). During the measurement, the rats were placed in a fixed container for 5–10 min. After the emotion was stabilized, the Ocuff and VPR sensors were sequentially placed on the base of the tail, and the tail of the rat was placed on a heating plate. When the blood flow reached the sensor threshold, the operation interface displayed the sensor pressure curve and blood pressure value. Five stable blood pressure measurements were performed, and the systolic blood pressure, diastolic blood pressure, and mean arterial pressure were recorded. The rats were maintained in a relaxed state throughout the procedure.

**Experimental method of Serum test (****Enzyme-linked immunosorbent assay testing):** The general methods of the experiment were as follows. We diluted the antibody, added the blocking solution dropwise, incubated the mixture at 37°C for 1–2 h, and washed. The sample was added after washing. The plate was sealed with a sealing film and incubated at 37°C for 1–2 h. After washing, antibody was added, and the sample was incubated again at 37°C for 1 h. After washing, the diluted enzyme conjugate working solution was added. Following incubation for 30 min, the sample was washed again. The reaction was terminated by adding the chromogenic substrate, and the result was evaluated.

**Experimental method of Immunohistochemistry***:* The general experimental methods were as follows: antigen retrieval was performed on the slices, and peroxidase activity was blocked. Subsequently, 3% bovine serum albumin was added to the histochemical circle to evenly cover the tissue. The primary antibody was added, and the sections were incubated overnight at 4 °C. Next, the secondary antibody was added, and the sections were incubated at room temperature for 50 min. Finally, diaminobenzidine was applied to develop color, and the nucleus was counter-stained. The sections were dehydrated and sealed. Thereafter, the integrated density of the target protein after staining was calculated.

The slices were analyzed with the Tissue Gnostics multi-spectral quantitative analysis system (TissueGnostics, Vienna, Austria), and the images were captured using a 20× high-powered lens.

**Experimental method of Immunofluorescence:** The general experimental methods were as follows: after antigen retrieval on the paraffin sections, the sections were blocked. The primary antibody was added, and the slices were placed in a humidified box and incubated overnight at 4°C. On day 2, the secondary antibody was added, and the nucleus was counter-stained with 4',6-diamidino-2-phenylindole staining solution. The autofluorescence quencher was added to the chemical circle for 5 min and rinsed with running water for 10 min. After mounting the slides, images were observed and captured under a fluorescence microscope (NIKON ECLIPSE C; NIKON, Japan). The Case Viewer software (3DHISTECH, Budapest, Hungary) analysis system was used to capture images for the immunofluorescence staining analysis. The ImageJ software (National Institutes of Health) was used for image analysis.

**Experimental method of Transmission Electron Microscope (TEM):** Myocardial tissue (1 mm^3^) was obtained from the apex of the heart and rapidly placed in the electron microscope fixation solution. Next, 1% osmium acid was prepared using 0.1 M phosphate buffer (pH 7.4) to fix tissues for 2 h in the dark. After dehydration at room temperature, infiltration and embedding were carried out. The samples were polymerized in an oven at 60°C for 48 h. The resin block was removed, and ultra-thin sectioning was performed at 60–80 nm prior to staining. Finally, a TEM (batch number: HT7800/HT7700; Hitachi, Japan) was used to observe and capture the images.

**Experimental method of WB**：This experiment was conducted as follows: total tissue proteins were extracted, and protein concentrations were determined. The protein was denatured and made into glue. Next, gel electrophoresis was performed, and the proteins were transferred from the gel onto nitrocellulose membranes. The second antibody was added after the primary antibody was incubated. Finally, the film was scanned and archived. ImageJ software (National Institutes of Health, USA) was used to analyze the integrated optical density of the target zone. Glyceraldehyde 3-phosphate dehydrogenase (GAPDH) was used as an internal reference. The relative optical density was used as the statistical analysis data.

**Experimental method of qPCR**

Before the experiment, all equipment (including pipette tips and centrifuge tubes) were sterilized to eliminate RNase. This experiment included the following three parts:

1. **Extraction of total RNA:**

Apical tissue (100 mg) was taken, added to the homogenization tube, and ground thoroughly. The slurry was centrifuged and the supernatant taken. Chloroform was added and centrifuged again. The supernatant was transferred to a new centrifuge tube, isopropanol was added, and it was placed at -20°C for 15 minutes. It was centrifuged again at 4°C. The white precipitate at the bottom of the tube was RNA. After the liquid was washed out, ethanol was added, centrifuged, the liquid was aspirated, and 15μl of RNase-free water was added to dissolve RNA. It was incubated at 55°C for 5 min. A Nanodrop 2000 was used to determine the RNA concentration and purity. After the blank of the instrument was set to zero, 2.5μl of the RNA solution was taken to be tested and placed on the detection base. The sample arm was put down and the absorbance detection was started using the software. RNA was diluted for samples with an excessively high concentration to a final concentration of 100-500 ng/μl.

1. **Reverse transcription:**
2. The reverse transcription reaction system was configured as follows:

| **Component** | **Volume** |
| --- | --- |
| 5 x Reaction Buffer | 4 μL |
| Oligo (dT)_18_ Primer (100 μM) | 0.5 μL |
| And Random Hexamer primer (100 μM) | 0.5 μL |
| Servicebio**^®^**RT Enzyme Mix | 1 μL |
| Total RNA ^*^ | 10 μL |
| RNase free water | Add to 20 μL |

2) The solution was mixed gently and centrifuged.

3) The reverse transcription program was set up as follows:

| **Temperature** | **Time** |
| --- | --- |
| 25℃ | 5 min |
| 42℃ | 30 min |
| 85℃ | 5 sec |

**3. Quantification of PCR**

1) PCR tubes (0.2 ml) were used to prepare the following reaction system. Three tubes were prepared for each reverse transcription product.

2× qPCR Mix 7.5μl

2.5μM gene primer (upstream + downstream) 1.5μl

Reverse transcription product (cDNA) 2.0μl

ddH_2_O 4.0μl

2）PCR amplification

| Stage1 | Stage2（40 cycles） | Stage3（Melting curve） |
| --- | --- | --- |
| 95℃，10min Predenaturation | 95℃, 15s denaturation | 65℃→95℃ |
|  | 60℃, 30s Annealing/Extension | Fluorescence signal is collected every time the temperature is increased by 0.5℃ |

1. **Result processing: ΔΔCT method:**

A=CT (target gene, **sample to be tested**)-CT (internal standard gene, sample to be tested).

B=CT (target gene, **control sample**)-CT (internal standard gene, control sample)

K=A-B

Expression multiple=2**^-K^**

The internal control GAPDH was used for standardization, and the relative expression of genes was calculated by the 2^-ΔΔCT^ method.
